# The biopsychosocial model of schizophrenia and cancer: Unraveling the etiopathogenesis of complex diseases

**DOI:** 10.1192/j.eurpsy.2022.2349

**Published:** 2022-12-15

**Authors:** Andrea Fiorillo, Antonio Giordano

**Affiliations:** 1 Department of Psychiatry, University of Campania “L.Vanvitelli”, Naples, Italy; 2 Sbarro Institute for Cancer Research and Molecular Medicine and Center of Biotechnology, College of Science and Technology, Temple University, Philadelphia, Pennsylvania 19122, USA; 3 Department of Medical Biotechnologies, University of Siena, Siena, Italy

Schizophrenia and cancer are complex disorders causing severe impairment and premature mortality. They both include a wide range of different illnesses, with different symptoms, course, and outcome. Although the causes of schizophrenia remain largely unknown, research into the etiopathogenesis of cancer has led to clarify its main genetic and environmental factors [1].

Our understanding of the etiopathological mechanisms of schizophrenia is still far from being conclusive. It is now conceptualized as a neurodevelopmental disorder, lying on a continuum, from mild psychotic experiences observed in the general population to frank psychotic episode.

Our diagnosis of schizophrenia is mainly based on non-observable signs and symptoms reported by patients, that are associated with various degrees of disability and that last for at least 6 months [2]. One of the most accepted theories of schizophrenia is the stress-vulnerability model [3], according to which psychotic symptoms would be based on a genetic or biological vulnerability, and triggered by stressful environmental factors [4]. This model is based on the biopsychosocial model of modern medicine.

Cancer follows a similar pathway. Several genetic loci have been identified to be responsible for the liability to the illness, and several biological (e.g., hormones), social (e.g., education), environmental (e.g., pollution), or behavioral (e.g., physical activity) factors either protect against or prompt the illness. This approach has opened the way to the personalized approach in cancer (e.g., breast cancer), giving significant hope to the patients and their families, and helping to destigmatize the illness. In mental health, although the role of environmental and social factors is now well established, biological and genetic studies are still far to get to definitive conclusions, as in oncology.

The etiopathological model of schizophrenia has much in common with that of cancer. First, the definition. Although a single word (cancer or schizophrenia) defines both disorders, we all know that they are umbrella terms covering several different types of disorders. Second, the staging system, which is well established in oncology and is becoming popular in psychiatry as well. Third, they (schizophrenia and cancer) share several risk factors, such as pollution, smoking (either nicotine or cannabis), migrant status, adverse life events, bullying, physical abuse and child maltreatment, and alcohol consumption. However, studies in oncology and psychiatry have consistently showed that these risk factors alone are not sufficient for initiating the disease (cancer or schizophrenia), but they must interact with other biological and social factors to determine the pathology.

Thus, the complexity of biological and psychosocial phenomena does not allow us to draw certain linear causal links. What is causal, that is, responsible for a cause–effect between two observable events, may appear casual, that is, the result of chance and randomness, as we are often unable to determine a direct relationship. In mechanisms as complex as cancer and schizophrenia, scientists often fail to discriminate between what is causal from what is random or chaotic.

The term “cancer” defines a group of extremely heterogeneous diseases (including more than 200 different types) with a multifactorial etiology. Both genetic and environmental factors (such as diet, viral infections, drugs, radiation, and pollution) contribute to the onset of cancer. At the genetic level, cancer is characterized by the progressive accumulation of multiple DNA mutations. The environment in which we live dictates the “rules” and determines when cancer will develop, through a sequence of unpredictable alterations, which might take many years.

The International Agency for Research on Cancer has identified many substances that are “definitely” carcinogenic to humans (118 agents) [5]. However, more than 800 substances belong to other categories (“probable carcinogens”, “possible carcinogens”, etc.), due to the limited number of available data or the fact that data in animal models were not confirmed in humans. Environmental pollution is one of these factors. Pollution is strongly affecting life in our planet. When we refer to pollution, we include lifestyle, such as diet, tobacco use, and alcohol consumption, but also radiation, infectious agents, and pollutants in air, water, and soil. However, although we still do not know whether these factors are also associated with the pathogenesis of schizophrenia, we now have enough data on the higher incidence of schizophrenia in people living under environmental circumstances which can have a common basis with cancer (e.g., air pollution, smoking, and alcohol).

If it is true that cancer is a heterogeneous, multifactorial “genetic disease of environmental origin,” in which genetic and environmental factors (chemical, physical, and biological) contribute, what can we say about schizophrenia? Is schizophrenia caused by a combination of genetic and environmental factors too?

We cannot exactly predict when and to which extent a certain environmental insult can cause the manifestation of a schizophrenic disorder or cancer, but we know that the combination of genetic alterations and environmental insults under certain conditions can lead to the onset of various pathologies [6]. If we look at migration studies, people migrating from high cancer risk areas (as well as high schizophrenia risk areas) to low cancer risk areas (or schizophrenia), or vice versa, in their lifetime assume the cancer rate of the country they move to [7], irrespective of the socioeconomic status.

Nongenetic factors, such as infections, childhood maltreatment, cannabis use, alcohol, smoking, and air, water, and soil pollution, can make the genome more “instable,” modifying its structure. Thus, the environment would “trigger” complex and specific cellular pathways that, through epigenetic modification, can become transmissible from one generation to another [8].

These changes are not “random” but affect specific areas of the genome. A protracted genetic stress tends to produce, in the medium or long term, a systemic genomic activation, through progressive and global hypomethylation of DNA and/or hypermethylation of CpG islands (normally hypomethylated) of the promoter region of tumor suppressor genes.

In recent years, the polygenic risk score (PRS), consisting in the total number of risk alleles carried by an individual patient, weighted by the odds ratio associated with each allele as derived from previous genome-wide association study findings, has been associated with the risk of developing the disorder [9]. In particular, findings from the Psychiatric Genomics Consortium have confirmed that PRS accounts for approximately 7% of variation in risk for schizophrenia. In addition, individuals scoring in the top decile are approximately 15 times more likely to manifest the illness compared with those in the bottom decile [10].

The concept that environmental factors, to which each of us is involuntarily exposed, represent a negligible risk today is outdated, particularly in light of growing incidence of cancer and schizophrenia in children and adolescents.

Thus, there is the need for an integrated and multilevel approach, which integrates politics, health education, and an efficient health system.

These new data in cancer research on the relationship between gene and environment have opened the field for studying thousands of genes simultaneously and monitoring their expression as a result of specific environmental exposures. Thus, environmental pollution must be necessarily addressed, as in fact it could represent a real possibility of reducing the onset of cancer in conjunction with enhanced prevention. The hope is that the same model could be applied to the study of schizophrenia and that, one day, we will be able to open the “black box” of schizophrenia and finally find “the cause” in a genomic alteration caused by stressful environmental factors.
